# Rationalizing antibiotic prescribing for bacterial pneumonia in patients with reported penicillin allergy—a qualitative study

**DOI:** 10.1093/jacamr/dlaf035

**Published:** 2025-03-13

**Authors:** Xer Min Nicole Lau, Xinle Zhu, Dylan Sidhu, Jia Wei Bay, Yin Mo, Paul Anantharajah Tambyah

**Affiliations:** National University of Singapore, Yong Loo Lin School of Medicine, 10 Medical Dr, Singapore 117597, Singapore; National University of Singapore, Yong Loo Lin School of Medicine, 10 Medical Dr, Singapore 117597, Singapore; National University of Singapore, Yong Loo Lin School of Medicine, 10 Medical Dr, Singapore 117597, Singapore; National University of Singapore, Yong Loo Lin School of Medicine, 10 Medical Dr, Singapore 117597, Singapore; Infectious Diseases Translational Research Program, Yong Loo Lin School of Medicine (National University of Singapore), 10 Medical Dr, Singapore 117597, Singapore; Department of Infectious Diseases, National University Hospital, 5 Lower Kent Ridge Road, Singapore 119074, Singapore; ADVANCE-ID, Saw Swee Hock School of Public Health, National University of Singapore, Singapore 117549, Singapore; Nuffield Department of Medicine, Centre for Tropical Medicine and Global Health, Oxford OX3 7BN, UK; Mahidol-Oxford Tropical Medicine Research Unit, Faculty of Tropical Medicine, Mahidol University, Salaya, Nakhon Pathom 10400, Thailand; Infectious Diseases Translational Research Program, Yong Loo Lin School of Medicine (National University of Singapore), 10 Medical Dr, Singapore 117597, Singapore; Department of Infectious Diseases, National University Hospital, 5 Lower Kent Ridge Road, Singapore 119074, Singapore

## Abstract

**Background:**

Penicillin allergy is commonly reported, yet often mislabelled. Such a label is associated with adverse outcomes in bacterial pneumonia. Despite recognition of the overlabelling of penicillin allergy and the awareness of potential adverse effects, there are limited data on the rationale for the management of patients with bacterial pneumonia and concomitant penicillin allergy.

**Objectives:**

To investigate the rationale guiding antibiotic prescription for bacterial pneumonia patients with reported penicillin allergy to improve outcomes.

**Methods:**

Semi-structured interviews were conducted between May and September 2022 to explore the management of patients with bacterial pneumonia and concomitant penicillin allergy. Data were analysed thematically using NVivo software. Recruitment was stopped when thematic saturation was reached.

**Results:**

Twenty doctors from the National University Hospital System, Singapore were interviewed. Role models and guidelines were found to be important in helping junior doctors make appropriate prescribing decisions. The ease of accessibility of detailed descriptions in allergy records and allergy services were among the concerns raised.

**Conclusions:**

Locally adapted approaches can be taken to appropriately label and delabel penicillin allergy, optimizing treatment and outcomes for patients with pneumonia and other common infectious diseases. We found that in an Asian urban context, role models and guidelines have been helping junior doctors make appropriate prescription decisions. More resources may be channelled into delabelling penicillin allergy to optimize patient outcomes.

## Introduction

Penicillin allergy is one of the most commonly reported antibiotic allergies globally, with 10% of adults reporting penicillin allergies in the USA.^1^ However, clinically significant IgE-mediated or T lymphocyte-mediated penicillin hypersensitivity is rare, with a reported prevalence of less than 5%.^1^ A Singapore study found that 85.8% of patients who reported a β-lactam allergy were found not to be allergic on specific testing.^2^

In Singapore, pneumonia is the fourth most common cause of hospitalization, accounting for 2.6% of discharges in 2021, and the second most common cause of death, being responsible for 20.7% of deaths even before the COVID-19 pandemic in 2019.^3^ Penicillin allergy may lead to the use of broader-spectrum antibiotics for bacterial pneumonia, which may have greater adverse effects, are less efficacious, and could contribute to the rise of antimicrobial resistance.^4^ A retrospective cohort study conducted in Pennsylvania^5^ found that patients admitted for bacterial pneumonia who were labelled with penicillin allergy had higher risks of hospitalization, acute respiratory failure, intubation, need for intensive care and mortality. A pilot study done by our team showed that penicillin allergy was associated with a higher severity of bacterial pneumonia. An abstract of this pilot study was accepted by the 27th Congress of the Asian Pacific Society of Respirology, November 2023. The negative impact of reported penicillin allergy on outcomes for these patients is a concern.

The adverse effects of penicillin allergy labelling are complex and multifactorial. Despite recognition of the overlabelling of penicillin allergy and the awareness of potential adverse effects,^6^ there are limited data on physicians’ rationale for the management of patients with penicillin allergy. There have been qualitative studies that investigated the views and understanding of penicillin allergy for primary care physicians (PCPs),^7^ but none explored the prescribing rationale in depth, and may not be applicable to a hospital setting, where prescribing patterns may be more heterogeneous.^8^ Multiple other studies also focus on exploring perceptions on the act of delabelling,^9^ or reporting allergies;^10^ there have not been any studies focusing on the management of the infections in penicillin-allergic patients. Understanding the motivations and thought processes guiding antibiotic prescribing by all levels of prescribers presents an opportunity for improvement in patient outcomes. This has not been well reported in the literature, especially for pneumonia. Recent developments in exploring the role of non-allergy healthcare professionals in penicillin allergy delabelling has proven to reduce the rates of spurious penicillin-allergy labelling.^11^

In Singapore, when a patient self-reports a penicillin allergy at first contact with the healthcare system, this is entered in the local hospital’s electronic health records system. The healthcare provider then fills an electronic form with multiple questions detailing the allergy, such as type of reaction to the medication, onset of reaction from time of administration or consumption of the drug. Details such as prior exposure to the drug or adverse reactions from drugs in the same pharmacological class are recorded if known.

Once recorded, the data are incorporated in the national electronic medical record system’s Critical Medical Information Store (CMIS), which will be visible to all healthcare providers regardless of which healthcare network they are practising in. When ordering any medication for the patient, the hospital’s electronic medical record, which is linked to CMIS, will generate a pop-up alert stating the patient’s reported allergy. At this point, the provider has the option to accept or override this alert before proceeding with prescription. These systems are used nationwide in Singapore, where this study was conducted.

We conducted a qualitative study to better understand the decision to prescribe antibiotics for patients labelled as penicillin allergic, particularly in an acute setting, focusing on the current state of management of patients with reported penicillin allergy and improving management of patients with reported penicillin allergy and delabelling. We used a descriptive qualitative approach as there are very limited qualitative data on antibiotic prescribing^12^ and thus a descriptive approach would be needed to guide the use of any theoretical framework.

## Methods

### Study design

We conducted a qualitative study using semi-structured interviews. Purposive sampling was first employed, recruiting Infectious Diseases and Rheumatology clinicians. Snowball sampling was then used to recruit additional participants from this initial group of participants. Recruitment was stopped when thematic saturation was reached. We included doctors who were currently or previously had been involved in the care of patients with bacterial pneumonia.

E-mails with information on this study were sent out to doctors working at the National University Hospital (NUH). The National University Health System consists of a major tertiary level hospital (1200 beds) and two regional general hospitals. Ideally, interviews were done in person, but online interviews via Zoom were also conducted when there was tightening of safe distancing measures due to the COVID-19 pandemic or if a suitable timing for in-person interviews could not be found.

Ethics approval was obtained from the National Healthcare Group Domain Specific Review Board which governs research in NUHS (Reference number 2021/00429). Written informed consent was obtained from all participants prior to each interview.

### Data collection

Interviews of approximately 30–60 min were conducted between May and September 2022.

We crafted our questions based on the gaps identified in existing literature and created an interview guide questionnaire with topics included, but were not limited to: past encounters with bacterial pneumonia patients and concomitant penicillin allergy; perceptions and attitudes towards management of these patients; and awareness and attitudes towards guidelines regarding treatment of bacterial pneumonia including scenario questions.

The full set of the interview questions is included in Table S2 (available as Supplementary data at *JAC-AMR* Online). Interviews were recorded using devices loaned from the National University of Singapore Center for Medical Education (NUS CenMED), transcribed verbatim and anonymized. There were three female (X.M.N.L., X.Z., J.W.B.) interviewers and one male interviewer (D.S.).

### Data analysis

Interviews were conducted and transcribed by study team members (D.S., X.M.N.L., X.Z., J.W.B.). Preliminary coding and analysis of the transcripts was done individually and independently using NVivo 11 software (QSR International, Burlington, MA, USA). Three cross codings were then done for each transcript and the codes were eventually summarized into themes. Recruitment was stopped when data analysis showed repetitive findings and reached thematic saturation at a sample size of 20 doctors.

We analysed the data thematically using Braun and Clarke’s six-stage method,^13^ with deductive coding based on literature findings and inductive codes generated from data. All participants spoke English, and interviews were audio-recorded and transcribed verbatim using Otter.ai (Mountain View, CA, USA).

The consolidated criteria for reporting qualitative research (COREQ) checklist is shown in Table S3 to ensure that the study is comprehensively reported.

## Results

The participant demographics are shown in Table 1. We interviewed 20 doctors from the National University Hospital System. There were 7 female doctors (35%) and 13 male doctors (65%) from different specialties and positions. The majority of our participants were from medical-related specialties and subspecialties, such as internal medicine (9; 45%) and rheumatology (3; 15%), since doctors in medical specialties treat bacterial pneumonia more often than doctors in surgical specialties. Their range of clinical work experience ranged from 1 to almost 30 years, as we gathered information from both junior doctors and senior consultants. The duration of interviews ranged from 24 to 51 min, with a mean duration of 38.7 min.

**Table 1.** Participant demographics

Demographic characteristics	No. of participants (*N* = 20)
Gender	
Male	13
Female	7
Basic medical training	
Singapore	10
UK	6
Ireland	1
Australia	3
Years of practice	
1–4	10
5–9	3
10 or more	7
Current position	
Medical officer^a^	3
Resident	7 (1 senior resident, 6 junior residents)
Consultant	5 (2 associate consultants, 3 consultants)
Senior consultant	5

^a^In the Singapore healthcare system, a medical officer is a Year 2 postgraduate and beyond, who may or may not have entered residency training yet.

Results would mainly comprise the current state of management of patients with reported penicillin allergy (see Section 1), followed by nuanced suggestions or measures in improving management in these patients (see Section 2).

### Section 1: Current state of management of patients with reported penicillin allergy

#### ‘First, do no harm’ principle governs antibiotic choice for penicillin allergies

When a reported penicillin allergy alert shows on the electronic prescribing system, most participants report taking extra care when prescribing antibiotics for such patients. This is usually due to concern over triggering allergic reactions when prescribing antibiotics that have cross-reactivity with penicillins (Table 2; Quote 1).

**Table 2.** Quotes relating to Theme 1.1

Theme 1.1: Reported penicillin allergy almost always changes decisions on antibiotic choice due to the fear of causing harm despite the impression that many are over-reported. ‘First do no harm’ is an often-repeated mantra.	
Quote 1	*‘Because there is still [the] possibility of cross reactivity with β-lactam, there is still a chance of allergic reaction with other β-lactams and sometimes these allergic reactions can be life-threatening and severe, such as anaphylaxis. That’s why I am always careful.’ Participant 16 (Associate Consultant)*
Quote 2	*‘…because it’s already reported, we won’t give them the [penicillin] antibiotics just to see if it’s just an adverse drug reaction or if it’s a true allergy.’ Participant 4 (Resident)*
Quote 3	*‘In truth, I think penicillin allergies are over-reported and no one really does anything about it in the acute setting…If there is an alternative of a different class, I find that people will just gravitate to the different classes of antibiotics.’ Participant 2 (Consultant)*

While doctors recognize that some reported penicillin allergies may not be true allergies, some are still uncomfortable with prescribing penicillin and would prefer to provide alternative options for these patients where available (Table 2; Quotes 2 and 3).

#### Penicillin-allergy alerts can improve patient safety but may be uninformative, leading to more conservative decisions on antibiotic prescribing

Electronic allergy alerts are often thought of as important safety nets in patient care to reduce preventable accidents (Table 3; Quote 6).

**Table 3.** Quotes relating to Theme 1.2

Theme 1.2: Uninformative penicillin allergy alerts lead to more conservative decisions on antibiotic prescribing.	
Quote 4	*‘…more than half of juniors will not check what the allergy is since additional steps [are] needed via accessing [the] system to see the reaction. The additional step renders people lazy.’ Participant 2 (Consultant)*
Quote 5	*‘The system doesn’t show what the allergy is so we don’t really know what the reaction is; sometimes it might not even be true allergy. This limits antibiotic choices and increases the susceptibility of patients to multidrug-resistant organisms.*’ *Participant 19 (Medical Officer)*
Quote 6	*‘…but it is always better to have [an] alert than not have it, so that you don’t go ahead and order something that might send someone to anaphylaxis.’ Participant 1 (Medical Officer)*

However, it may sometimes be more of a nuisance, and participants highlighted the lack of practical impact in daily practice. Inherent shortcomings of the pop-up alert includes absence of allergy reaction description and verification. Coupled with the additional clicks needed to access the national allergy reporting system, i.e. CMIS from local hospital electronic medical records, this results in a non-streamlined prescription process where key information has to be separately pieced together by the doctors (Table 3; Quotes 4 and 5).

#### Personal experience and knowledge determine antibiotic choice in general in the absence of systemic guidelines

The antibiotic choice for patients with reported penicillin allergy and bacterial pneumonia is dependent on medical hierarchy as juniors commonly default to the antibiotic choice of the senior doctor, such as the consultant or senior consultant (Table 4; Quote 7).

**Table 4.** Quotes relating to Theme 1.3

Theme 1.3: Personal experience and knowledge determine antibiotic choice in general in the absence of systemic guidelines	
Quote 7	*‘‘I will suggest but won’t impose because I will usually just follow what the consultant in charge orders.’ Participant 4 (Resident)*
Quote 8	*‘…ultimately they assume overall responsibility for the patient so their name is on the discharge summary, not mine, so if after discussion they still prefer the antibiotic A over B, then it is their call.’ Participant 3 (Resident)*
Quote 9	*‘[I would] mostly [interact with] juniors, and it is every day, usually telling them to dial back the antibiotics. Usually they have escalated to meropenem or something [like that] all the time.’ Participant 15 (Senior consultant)*
Quote 10	*‘But realistically speaking, if you are in the on-call situation, you are by yourself so [you] need to make [the] decision by yourself. [You] may not have much senior support. But if [the decision occurs] during the day, if they want to give advice and what antibiotic to change, sure.’ Participant 3 (Resident)*
Quote 11	*‘Standard ones [cases of bacterial pneumonia] [are] just treat[ed] by ourselves, assuming we are on call without the registrar or consultant.’ Participant 8 (Resident)*
Quote 12	*‘Usually I don’t ask [seniors], only during rounds I will tell the consultant about the antibiotics that I started. But on call, I will start antibiotics myself.’ Participant 14 (Resident)*
Quote 13	*‘If I was on call and seeing this patient, I would start broad-spectrum [antibiotics] with something safe first. And then if I was the morning team, then maybe I would [do a] drug challenge [for] the patient…you have more manpower available in case something happens [during the drug challenge].’ Participant 9 (Resident)*
Quote 14	*‘Antibiotic prescription is based on experience. [It] depends on how much they feel that they own the issue of antibiotic prescribing.’ Participant 12 (Consultant)*

One reason for this could be the lack of junior doctor ownership of medical care in public hospitals. Although junior doctors are able to create medication orders, most legal documents such as discharge summaries bear the senior doctor’s name as the doctor-in-charge of the patient. This contributes to the practice of deferring to the antibiotic choice of the consultant (Table 4; Quote 8).

One justification for this dependence on hierarchy is the lack of clinical experience in junior doctors. As shared by a senior doctor, inaccurate antibiotic choices are often made by juniors and thus have to be corrected by senior doctors (Table 4; Quote 9).

However, during evening shifts when senior doctors are usually not around, junior doctors would make the initial antibiotic choice themselves whenever the patient is admitted overnight. They would then proceed to present the patient during morning rounds and highlight their antibiotic choice, which might be changed depending on the senior doctor’s opinion (Table 4; Quotes 10–12).

Generally, junior doctors prefer to start what they perceive to be safer or broader-spectrum antibiotics if a patient with penicillin allergy was admitted overnight, rather than performing a drug challenge after hours. This reinforces the prevalent ethos of first doing no harm (Table 4; Quote 13).

In general, personal experience was then revealed to be a determinant in the level of comfort when prescribing antibiotics. With repeated encounters of the same patient group, more commonly experienced by senior clinicians, confidence and knowledge in handling antibiotic prescription grows (Table 4; Quote 14).

### Section 2: Improving management of patients with reported penicillin allergy

#### Importance of a judicious antibiotic stewardship programme

Reported penicillin allergy can sometimes lead to prescription of broad-spectrum empirical antibiotics, creating antibiotic stewardship issues (Table 5; Quotes 15 and 16).

**Table 5.** Quotes relating to Theme 2.1

Theme 2.1: Importance of a judicious antibiotic stewardship programme	
Quote 15	*‘…I do sometimes call out people who escalate antibiotics to Tazocin for no good reason; sometimes I feel it’s so unnecessary, which is a bit frustrating sometimes…we use antibiotics like Tazocin far too often. And sometimes I feel [that] this is not very good antibiotic stewardship as well.’ Participant 1 (Medical officer)*
Quote 16	*‘I think the patients with penicillin allergy tend to end up getting a lot of IV medications, even though they are not very unwell. Those with no allergies tend to be able take oral antibiotics more easily and so they tend to not stay for a very long time.’ Participant 6 (Senior consultant)*
Quote 17	*‘The other person who may provide input before you ask for it is actually the antibiotic stewardship programme (ASP). In our hospital we have this team with Infectious Disease specialists and pharmacists who run this ASP that will review antibiotic prescriptions.’ Participant 2 (Consultant)*
Quote 18	*‘Since the patients tend to get IV, the heavy guns [stronger antibiotics] are then used and we end up having to justify that because the Infectious Disease doctors have an antibiotic stewardship programme. For patients on very broad-spectrum antibiotics, they [doctors from Infectious Diseases] would then pose questions and suggest de-escalation of the antibiotics.’ Participant 6 (Senior Consultant)*

Antibiotic stewardship teams led by pharmacists and Infectious Disease specialists in the hospital regularly monitor prescription of broad-spectrum antibiotics and report these to the Ministry of Health (MOH) (Table 5; Quotes 17 and 18).

Such programmes are limited by their need for a significant time commitment by team members but they do provide guidance to junior doctors and non-specialists and are well established in Singapore hospitals.^14^ While they provide some generic guidance for patients labelled as penicillin allergic, their main focus lies in reducing antimicrobial resistance by targeting antibiotic prescription generally.

#### Need for a system-level guideline

As experience takes years to develop, the majority of participants highlighted the benefits of institutional guidelines across all levels of seniority. These were designed for antibiotic prescription and include alternative antibiotics recommended for patients with reported penicillin allergy, based on the site of infection (Table 6; Quotes 19 and 20).

**Table 6.** Quotes relating to Theme 2.2

Theme 2.2: Need for a system-level guideline	
Quote 19	*‘I think that [they] are very helpful, most of the time they are up to date, which is very helpful because up until recently, people were still giving Tazocin for any healthcare-acquired pneumonia even though I think recent research shows that Augmentin is [has] good coverage. ‘Participant 1 (Medical officer)*
Quote 20	*‘I think that they [the guidelines] are good actually. They cover important things, like they cover melioidosis for severe CAP. ‘Participant 15 (Senior Consultant)*
Quote 21	*‘…problem is they [research papers] are not necessarily easy to read or digest. If you are trying to get through it quickly in [a] bite-sized way, [it] may not be the most efficient way in taking in information… That’s where different sources come in where people try to, I think, package information and curate information by selecting the most important aspects and highlights and presenting it in a way that fosters in memory and retrieval’ Participant 10 (Consultant)*
Quote 22	*‘If there is new evidence before guidelines can be changed, if the department [the Department of Emergency Medicine] is aware of it, usually colleagues will share [it], we will appraise it to see if we should adopt it.’ Participant 20 (Senior Consultant)*
Quote 23	*‘I keep up [with] the literature through a few free open-access journals, medical education sources, [and] paid subscription for [an] emergency medicine educational podcast. Departmental Mortality and Morbidity Rounds [and] grand ward rounds also keep us updated too.’ Participant 10 (Consultant)*

However, a participant also shared that such international guidelines may be underutilized as it may be difficult for readers to pick out key points. Such information becomes easier to remember when a third party repackages it (Table 6; Quote 21).

Although individuals update their clinical practice based on the current literature; these are not the same as published authoritative guidelines, which need to be up to date (Table 6; Quotes 22 and 23).

#### Delabelling allergies in patients may be difficult in an acute setting

A consultation service is offered by the Division of Rheumatology’s allergy specialists to delabel patients with penicillin allergy whose allergy cannot be objectively confirmed from the electronic alert. With delabelling, utilizing β-lactam antibiotics would be a safe and reliable option where clinically relevant in the future care of patients, thus having the potential to improve overall patient outcomes (Table 7; Quote 25).

**Table 7.** Quotes relating to Theme 2.3

Theme 2.3: Delabelling	
Quote 25	*‘So in the allergy clinic, we see patients with penicillin allergies who can be referred to us from various sources but primarily the intent is to work out if they have [a] penicillin allergy. So we take a detailed history and go on to offer skin testing then drug challenge test. If the patient passes all of these components, then [he/she is] unlikely to have true penicillin allergy, then [he/she] can be prescribed penicillins in the future for any infection.’ Participant 13 (Senior Consultant)*
Quote 26	*‘Best practice is to refer to penicillin allergy clinic but [I] don't really know how many people will actually do it, as it is [for] a short course [of antibiotics]…[There is a] high threshold to challenge patients in situations that are impossible to be [a] true allergy, but otherwise [I] would refer [patients] to dermatology or allergy’ Participant 12 (Consultant)*
Quote 27	*‘If a patient develops a rash after taking penicillin, it’s usually labelled as an allergy without formal testing. We don’t routinely send patients with an allergy for allergy testing.’* ‘*Outpatient [allergy clinic]—by right, all these patients should be seen, but the service is very very busy so [I’m] not sure whether it is really done ‘Participant 19 (Medical officer)*

Although this service is available, there are significant logistical constraints, making it difficult for physicians to ensure timely delabelling. Participants also questioned the risks of drug challenge for antibiotics that are only prescribed for a brief duration, which is the case for most patients with bacterial pneumonia compared with chronic or recurrent infections (Table 7; Quotes 26 and 27).

## Discussion

Through in-depth interviews of key persons involved in antibiotic prescribing in a tertiary hospital setting, we found that key factors, including seniority and accessibility, which are not frequently cited in the literature, may have a significant role in Singapore and other parts of Asia. Furthermore, we found that the acute nature of bacterial pneumonia might serve as a barrier to delabelling allergies.

Consistent with our qualitative results, while junior doctors may write the prescription, the final decision frequently lies with senior doctors. This issue is probably reflective of the whole postgraduate ecosystem in Singapore and many other countries where junior doctors often feel less empowered or are under stress.^15^ Senior doctors could incorporate teaching about penicillin allergies during ward rounds or potentially be available after hours.^16,17^

Prescriber behaviour is shaped by clinical guidelines and while such guidelines in managing penicillin allergies are undoubtedly helpful in providing an overarching framework, they may not fully address intangible factors such as risk aversion, commonly seen in junior doctors. With seniority also comes experience in handling patients with allergies, and hence nuanced decision-making during antibiotic prescription. In an effort to foster change, it is important to bridge the gap between guideline-suggested practice, e.g. allergy testing and clinical practice, for prescription behaviours to shift over time. Educational interventions from seniors teaching juniors could help build confidence in managing penicillin allergies, integrating allergy testing into routine care for patients and addressing the anxiety currently seen in junior staff when challenging penicillin allergies. Examples could include training on tools to identify low-risk patients for delabelling, safety and efficacy of allergy testing.

The electronic medical record helps to reduce potentially lethal medication errors.^18^ However the unintended consequence of a broad allergy alert system is the risk that all drug reactions will be flagged without sufficient details, leading to potentially inappropriate prescribing. Although electronic allergy alerts theoretically reduce the risk of prescribing the wrong antibiotics, our informants did indicate this concern about alerts and the limitations in the current system, where multiple clicks are required to obtain more details of allergic reactions (i.e. which are more likely to be true anaphylaxis) from the national medical record system or CMIS.^19^ In a busy clinical setting, the lack of nuance and difficulty of access may cause overprescription of alternative antibiotics to penicillin-allergic patients. While information on the system is deliberately limited to protect medical confidentiality, perhaps with artificial intelligence, targeted release of information on the nature of drug allergies could be made available to physicians, especially after hours, to enhance efficiency and accessibility.

In a similar vein, the acute setting of such infections may also serve as a barrier to delabelling. Our hospital has a state-of-the-art allergy testing facility but our participants suggest that the demand is likely to outstrip capacity. Most doctors may also not consider delabelling patients for a short course of antibiotics. Creative solutions are needed to triage patients labelled with penicillin allergy to optimize the use of allergy testing services. One also wonders if delabelling would be more commonplace in a less busy service with fewer budget constraints, such as those in Singapore’s public healthcare setting.

Programmes for delabelling penicillin allergy have been implemented in countries such as the UK and Australia.^20^ Programmes like these have been shown to increase appropriate prescription while reducing adverse events of mislabelled penicillin allergies. The British Society of Allergy and Clinical Immunology (BSACI) has developed a guideline for penicillin allergy delabelling services by non-allergists.^21^ A recent randomized clinical trial in Australia and North America also showed that this could be done safely and efficiently.^22^

Our findings also emphasized the importance of guidelines, both local and international. Easing access to guidelines, as well as detailed allergy data, is likely to be beneficial. As mentioned by a participant, information from evidence-based sources may not be easily committed to memory. It is possible that time constraints may be a factor in this. By making guidelines easily digestible for time-pressed clinicians, it may better enhance their application in real life.

Limitations of the study include the fact that it is a single-centre study, as well as the use of snowballing recruitment and the voluntary nature of subject selection, which may have contributed to bias. Our results may not be representative of those who did not choose to participate and those working in other hospitals, due to a potential difference in behaviour patterns. Though our study reached thematic saturation, due to the limited scope of the study encompassing one hospital system, our study results may not be generalized to different settings due to sample characteristics and context differences. Hence it is important for further studies to be conducted to prevent drawing firm conclusions from these series of interviews. Given that approximately half of the 20 individuals interviewed were in residency, mainly junior doctors, this large heterogeneity renders interpretation of data and suggestions of solutions challenging as there may be knowledge gaps due to insufficient training. A potential area for future studies would be to investigate if results were different for those who have not completed training versus those who were more advanced.

In conclusion, we found that in our Asian urban context, role models and guidelines are both important in helping junior doctors make appropriate prescribing decisions in patients with pneumonia and penicillin allergies. Ease of access to electronic and clinical services could potentially improve delabelling of penicillin allergy to optimize outcomes for patients with pneumonia and other common infectious diseases. In addition, there could be greater promotion of delabelling services, and hospitals could implement policies to encourage clinicians to make greater use of such services.

## Supplementary Material

dlaf035_Supplementary_Data

## Supplementary data

Tables S1 to S3 are available as Supplementary data at *JAC-AMR* Online.
