# Supplementary material for: Rationalizing antibiotic prescribing for bacterial pneumonia in patients with reported penicillin allergy—a qualitative study
Source: JAC Antimicrob Resist. 2025 Mar 13;7(2):dlaf035. doi: 10.1093/jacamr/dlaf035 (PMC11904779; doi:10.1093/jacamr/dlaf035)
Supplement: dlaf035_Supplementary_Data [file dlaf035_supplementary_data.docx]

**Supplementary Material**

**Table S1. Participant Demographics**

| **Demographic Characteristics** | **No. of participants (n = 20)** |
| --- | --- |
| *Gender* | |
| *Male* | 13 |
| *Female* | 7 |
| *Basic medical training* | |
| Singapore | 10 |
| UK | 6 |
| Ireland | 1 |
| Australia | 3 |
| *Years of practice* | |
| *1-4* | 10 |
| *5-9* | 3 |
| *10 or more* | 7 |
| *Current position* | |
| Medical officer | 3 |
| Resident | 7 (1 senior resident, 6 junior residents) |
| Consultant | 5 (2 associate consultants, 3 consultants) |
| Senior consultant | 5 |
| *Current specialty* | |
| Internal Medicine | 9 |
| Infectious Diseases | 1 |
| Rheumatology | 3 |
| Medical Oncology | 1 |
| Anaesthesia | 1 |
| Dermatology | 1 |
| Intensive Care | 1 |
| Emergency Medicine | 2 |
| Paediatrics | 1 |

**Table S2. Qualitative Interview Questions**

| Q/N | Question |
| --- | --- |
| **General Particulars** | |
| 1 | How long have you been practising medicine? |
| 2 | What is your current position? |
| 3 | What specialty do you work in? |
| 4 | Could you share about what you do in your posting/position? |
| 5 | Tell us more about other clinical settings you have worked in? |
| **Questions about Past Encounters** | |
| 1a | How have you been involved in treating patients with bacterial pneumonia before?   - When  and which setting were you involved in this?  For instance,  was it in the past or are you still treating such patients now? |
| 1b | If you are able to recall, could you describe the treatment for these patients with bacterial pneumonia? In particular, how did you decide on the antibiotics to use?  Did you ask a senior or do you have a preferred antibiotic – what is your antibiotic of choice?  Do you use guidelines from hospital or professional societies? |
| 2a | Have you been involved in treating patients with concomitant penicillin allergy?   - What would happen if these patients use penicillin? - Was it a confirmed allergy? (e.g. face swelling)   *(If answer to 2a is no, skip to 3b)* |
| 2b | If you are able to recall, could you describe their treatment? (patients with concomitant penicillin allergy) |
| 3a | How did you come to decide on this particular treatment? (for patients with reported penicillin allergy with bacterial pneumonia?)   - What was the reason behind this treatment regime? - Are there specific steps or criteria you took? Did you refer to a guideline or ask a senior or someone else such as a pharmacist? - Have you ever had an alert pop up in the electronic medical order for drug allergy for someone you ordered an antibiotic for? How did you feel about that? What did you do next?. |
| 3b | Could you share with me how the management for patients with bacterial pneumonia and penicillin allergy differs from the usual treatment for similar patients without penicillin allergy? |
| **Questions about Perceptions and Attitudes Towards Prescription** | |
| 1a | What do you think about when prescribing a beta-lactam to patients with reported penicillin allergy?   - Is there any particular emotion that you feel when prescribing it? (e.g. confident, afraid) |
| 1b | Tell me more about your response. (reasons, etc.) |
| 2a | How often do you interact with colleagues both seniors and juniors, about their prescription habits?  Do you ask about antibiotic prescribing outside of working ward rounds?   - Do you interact with your colleagues about prescriptions for bacterial pneumonia? - If you require help, do you ask for advice from people? - Who do you consult with? (prompt: friend, specialist, senior) - Do you look at microbiology reports or do phone consults? |
| 2b | How do you think your colleagues – peers, seniors, juniors would feel about prescribing a beta-lactam to patients with reported penicillin allergy? |
| 2c | Why do you think they feel this way? |
| 3a | Under what circumstances would you prescribe a non-beta-lactam to a patient with reported penicillin allergy? |
| 3b | What would be your rationale in doing so? |
| 4 | Under what circumstances would you prescribe a beta-lactam to a patient with reported penicillin allergy? |
| 5 | Do you think there is a difference between suspected and confirmed bacterial pneumonia in terms of the diagnostic aspect?   - How would you define a confirmed bacterial pneumonia? Do not worry, there is no right answer! |
| 6 | What factors would you consider when choosing an antibiotic for a patient with reported penicillin allergy and **suspected** bacterial pneumonia |
| 7 | What factors would you consider when choosing an antibiotic for a patient with reported penicillin allergy and **confirmed** bacterial pneumonia? |
| **Questions about Awareness and Attitudes Towards Guidelines Regarding Treatment of Bacterial Pneumonia** | |
| 1a | Are you aware of any guidelines for the treatment of bacterial pneumonia?   - Latest Singapore national guidelines were updated in 2006, which have already expired. |
| 1b | *(If yes)* Which guidelines are you aware of? What do the guidelines say?   - International guidelines  such as the American Thoracic Society, European - National guidelines - Local guidelines (institution based)   *(If no)* What other sources are you aware of? (other than guidelines)   - E.g. Research papers, online sources (if so, which?), advice from seniors, medical textbooks, phone consult with Respiratory Medicine or Infectious Diseases |
| 1c | Are you using/following these guidelines/sources for the treatment of bacterial pneumonia? |
| 1d | *(If yes)* How do you feel about these guidelines/sources?  *(If no), Can you share with me more about your hesitance in using these guidelines?* |
| **Scenario Questions** | |
| Scenario 1 | A 20 year old male is admitted with lobar pneumonia. He has a history of a non-specific, non-urticarial rash (e.g. feathery or spidery rash) 3 days after taking amoxicillin 5 years ago. He is hemodynamically stable except a little tachycardic. Total wbc is 15K, CRP 100. COVID PCR negative. |
| 1a | What would you prescribe? |
| 1b | Why would you prescribe these medications? Can you walk me through how you decided on this course of treatment? |
| 1c | Would you change your management if he had facial swelling instead of the non-specific rash? |
| Scenario 2 | A 65 year old lady admitted with lobar pneumonia. She is borderline hypotensive. Sputum gram stain shows gram positive cocci in short chains. She has a history of vague allergic reaction to amoxicillin 40 years ago, but she cannot remember clearly. |
| 2a | What would you prescribe? |
| 2b | Why would you prescribe these medications? |
| **END OF SCENARIO QUESTIONS** | |
| 3a | Have you ever had patients with severe side effects from antibiotics used to treat bacterial pneumonia? |
| 3b | How would you decide what to change to?   - What would be your second line treatment? |
| 4a | What would you do if the patient had mild side effects? |
| 4b | *(If yes)* How would you decide what to change to?   - What would be your second line treatment?   *(If no or depends)* What factors have you considered which contributed to your hesitancy towards changing your management? |
| 5 | If you start a patient on a non-beta-lactam due to a reported penicillin allergy, what will make you change to a beta-lactam? |
| 6 | If pneumococcus is found in the sputum or blood and the patient has a reported penicillin allergy, would you change your treatment? Why or why not? |
| **Questions about empirical results of our study** | |
| 1 | How do you think the rate of mortality differs between non-penicillin allergic and penicillin allergic patients with bacterial pneumonia? |
| 2 | Our literature review/preliminary results/study found that the difference between mortality in non-penicillin allergic patients and penicillin allergic patients **was/was not** statistically significant. Why do you think this is so? |

**Table S3. Consolidated Criteria for reporting Qualitative Studies (COREQ): 32-item Checklist**

| **Domain 1: Research team and reflexivity** | | |
| --- | --- | --- |
| **Personal Characteristics** | | |
| 1. | Interviewer/ facilitator | BJ, DS, NL, ZX conducted all the interviews |
| 2. | Credentials | Year 5 medical students in National University of Singapore Yong Loo Lin School of Medicine (Class of 2024) (NUS YLL) |
| 3. | Occupation | Medical students of NUS YLL |
| 4. | Gender | Research team included female and male individuals. |
| 5. | Experience and Training | Interviewers had 0 years of previous experience in conducting qualitative research but were guided and trained by the principal investigator and experienced research doctors in the field of qualitative research. |
| **Relationship with Participants** | | |
| 6. | Relationship established | There was no personal relationship between interviewers and participants prior to data collection. When first contacting eligible participants upon mass invite through emails and snowballing sampling method, interviewers introduced themselves as medical students from NUS YLL under the mentorship of their Principal Investigator and answered any open questions prior to enrolling the participants and scheduling the interview. |
| 7. | Participant knowledge of the interviewer | Interviewers introduced themselves and the study to participants. This introduction included their overarching interest in the topic as well as the reasons and goals of conducting this research. |
| 8. | Interviewer characteristics | All the interviewers are Singaporeans, and this helped us understand the context of the Singapore healthcare system better, however it might have also resulted in some personal biases. As part of the systematic debriefings conducted throughout data collection and analysis stages, emerging risks of biases were discussed to mitigate the impact of them affecting the results of the study. |
| **Domain 2: Study Design** | | |
| **Theoretical Framework** | | |
| **9.** | Methodological orientation and Theory | The overarching study was guided by an action research design approach. Data collection for this manuscript was guided by thematic analysis approach.  *(see Methods section for further information on data analysis processes)*. |
| **Participant Selection** | | |
| 10. | Sampling | Snowballing sampling method was used and we ensured that the inclusion and exclusion criteria was met before enrolling the participants. |
| 11. | Method of approach | Participants were contacted via email addresses and mobile numbers which was provided to us when they indicated interest to participate in the study, whereby PDPA was upheld. |
| 12. | Sample size | 20 participants were interviewed in this study. |
| 13. | Non participation | There was 1 participant who pulled out of the study due to hospitalisation leave and unable to schedule a suitable time for the interview. |
| **Setting** | | |
| 14. | Setting of data collection | Interviews were held in NUS Yong Loo Lin School of Medicine buildings, NUHS Tower block meeting rooms when they were conducted physically.  However in view of the Covid-19 pandemic, an option of conducting interviews via an online platform i.e. Zoom was also available. |
| 15. | Presence on non participants | Participants were encouraged to join the online interview alone and from a space where it was convenient and felt comfortable for them to be in. |
| 16. | Description of sample | Characteristics of the sample size was reported in the manuscript. |
| **Data Collection** | | |
| 17. | Interview guide | Interviews were conducted following a piloted and refined semi-structured interview guides, focusing on participant’s personal experience with |
| 18. | Repeat Interviews | No repeat interviews were carried out. |
| 19. | Audio/ Visual recording | All interviews were audio recorded with prior consent from the interviewees. |
| 20. | Field Notes | Interviews took transcripts during and was re reviewed after the interviews which later served as the basis of discussion of findings. |
| 21. | Duration | Duration of interview was reported in the manuscript. Each interview is estimated to take 30-60 minutes. |
| 22. | Data saturation | Interviews were stopped once data saturation was achieved. |
| 23. | Transcripts returned | Transcripts were not returned to participants. |
| **Domain 3: Analysis and Findings** | | |
| **Data Analysis** | | |
| 24. | Number of data coders | The codebook was developed collaboratively by DS, NL, XL and JW with support of all authors. The entire dataset was then coded by DS, NL, XL and JW with regular discussions. |
| 25. | Description of the coding tree | The codebook was developed inductively based on the familiarisation of the interview and focused on developing themes on rationalising antibiotic prescription habits in patients with penicillin allergy. |
| 26. | Derivation of themes | Themes emerged inductively during transcribing of interview scripts, formulating of the code book and multiple rounds of synthesis into themes. |
| 27. | Software | Nvivo 11 (QSR International) was used for coding the data. |
| 28. | Participant Checking | Findings have not been discussed with participants. |
| **Reporting** | | |
| 29. | Questions presented | Participant quotations are presented verbatim throughout the results section. We use participant number and position as identifiers. |
| 30. | Data and findings consistent | We closely link our findings to our data throughout the results section. |
| 31. | Clarity of major themes | Major themes are discussed and clearly elaborated upon throughout the results section. |
| 32. | Clarity of minor themes | Minor themes and specific cases are discussed in the results section and case studies were presented in the discussion section. |
